# Accuracy of the London Atlas and RP Atlas in age estimation of Southern Brazilians

**DOI:** 10.4317/jced.63029

**Published:** 2025-10-17

**Authors:** Letícia Copatti Dogenski, Ademir Franco do Rosário Júnior, Vanessa Koltermann Sartori, Micheline S. Trentin, Juliane Bervian, Pedro Henrique Corazza, Yuri Dal-Bello, Matheus Albino Souza, Rodrigo Pimentel, Felipe Gomes Dallepiane, João Paulo De Carli

**Affiliations:** 1 PhD, postgraduate Program in Dentistry, University of Passo Fundo, Passo Fundo, Rio Grande do Sul, Brazil; 2 PhD, postgraduate Program in Dentistry, São Leopoldo Mandic, Campinas, São Paulo, Brazil; 3 MSc, postgraduate Program in Dentistry, Federal University of Santa Catarina, Florianópolis, Santa Catarina, Brazil

## Abstract

**Background:**

To assess the accuracy of the London Atlas and RP Atlas methods estimating age and diagnosing adulthood in a Southern Brazilian population.

**Material and Methods:**

A total of 1,099 panoramic radiographs from individuals aged 15.00-22.99 years were analyzed. The London Atlas and RP Atlas tools were used to estimate age based on the developmental stages of the upper and lower left third molars (teeth 28 and 38). Statistical analysis included Spearman's rank test, Bland-Altman plots, Lin's concordance coefficient, and ROC curves, with adulthood (&gt;18 years) as the cutoff (p&lt;0.05).

**Results:**

Both methods showed high accuracy, with the London Atlas exhibiting lower mean errors. Errors increased with age and were higher in females. The best adulthood cutoff for the London Atlas was 18.5 years for tooth 28 (78.5% accuracy) and 19.5 years for tooth 38 (80.0% accuracy). For the RP Atlas, the cutoff was 17 years for both teeth (80.8% and 80.0% accuracy, respectively). Both methods were suitable for age estimation and adulthood diagnosis, with the London Atlas tending to overestimate age - an advantage in legal contexts requiring caution.

**Conclusions:**

Validating age estimation methods in specific populations enhance accuracy, preventing errors when applying methods developed for other populations and ensuring suitability for the local context. Research that applies these methods can provide a foundation for adjustments to the Atlases and their application in diverse subpopulations, and even serve as the basis for the establishment of an international data repository.

## Introduction

Age estimation is one of the most widely used methods for investigating an individual's characteristics. In addition to assisting in forensic examinations and the formation of a biological profile of the deceased or the examination of living individuals, it can assess the probability that a person has reached a legally relevant age (1), in cases of adoption (2), asylum requests from refugees (3), absence or falsification of civil records (4), and criminal cases. Dentistry is involved in age estimation through bone and/or dental development, which consists of mineralization stages according to the phases of human development (5 , 6). Thus, it is possible to evaluate the chronology of tooth eruption or estimate the stage of crown and root mineralization of dental organs through radiographs (7), relating it to the individual's possible actual age. The accuracy of this estimation is higher during periods when teeth are actively developing, tending to decrease as mineralization is completed. Thus, in age estimations of young adults, the third molar is studied, as it exhibits later stages of mineralization compared to other teeth (8). For the assessment of dental mineralization, estimations based on Atlases can be used, as well as orthopantomograms, which provide a direct image comparison, resulting in a simplified, low-cost, easy-to-apply, and non-destructive approach that can be used for both living and deceased individuals (5 , 9 , 10). Among the available Atlases for dental age estimation, the London Atlas (5) stands out, developed based on the observation of a London population sample. Recently, Sousa et al. (10) also developed the RP Atlas, based on a population from southeastern Brazil. As with these, several methodologies have already been proposed in the literature (5 , 11 - 14), each developed for a specific population group. However, differences in accuracy may exist when applying a given technique for age estimation depending on the studied population, making it essential to apply and validate different methodologies across various populations (15). Thus, these studies are crucial to ensuring that, in cases where age determination is required, the appropriate methods are correctly chosen and applied to the specific population group. Considering this, the objective of this study is to evaluate the accuracy of the London Atlas and RP Atlas methods in estimating the age of a sample of individuals from southern Brazil, based on third molar images in panoramic radiographs.

## Material and Methods

This cross-sectional analytical observational radiographic study was approved by the Research Ethics Committee of the University of Passo Fundo (CEP/UPF, approval no. 5.603.446). - Sample The convenience sample consisted of 1,099 panoramic radiographs selected from the records of patients aged between 15.00 and 22.99 years who attended the Dentistry Program at the University of Passo Fundo (CO/UPF) between 2016 and 2022. The presence of teeth 28 and/or 38, with adequate radiographic visualization, was required. Panoramic radiographs with poor quality, including errors in image acquisition or processing, evident bone lesions, developmental disorders, or missing information regarding sex, date of birth, or the date of radiographic image acquisition, were excluded from the study. A retrospective review of patient records was also conducted to collect the following data: patient record number, date of birth, sex, and date of radiograph acquisition. Each record and radiograph was assigned a randomly generated numerical identification code, unrelated to the patient record number or any personal data, to ensure data confidentiality. The radiographs were acquired using an Eagle Digital device (Dabi Atlante, Ribeirão Preto, SP - Brazil) with settings of 75 kVp and 8 mA. The radiographic images were exported in JPEG file format, saved in high resolution on a computer, named with the numerical identification code, organized into folders according to the individual's sex, and exported to the Adobe® Photoshop® CC image editing software. In Photoshop, identification data were cropped using the software's cropping tool. Additionally, brightness, contrast, and zoom adjustments were made to enhance image visualization when necessary. - Evaluator calibration Two examiners underwent a training period for the application of the London Atlas and RP Atlas techniques, following the methodological guidelines. An Intraclass Correlation Coefficient (ICC) test was conducted, for which 10% of the panoramic radiographs from the final sample were randomly selected to include all age groups and sexes of the patients. The selected sample was evaluated by Observer 1 (L.C.D.) at two different time points, with a 45-day interval between them, and once by Observer 2 (V.K.S.). The intra-observer ICC (London Atlas: 0.961 (28) and 0.967 (38); RP Atlas: 0.979 (28) and 0.987 (38)) and inter-observer ICC (London Atlas: 0.930 (28) and 0.937 (38); RP Atlas: 0.965 (28) and 0.973 (38)) values were considered excellent (16). - Application of the London Atlas Method The London Atlas method (5) was applied using the "Data entry" option in the software provided by the authors on the Queen Mary University of London website (https://www.qmul.ac.uk/dentistry/atlas/software-app-full-width-/). This option allows users to define the sex (male, female, or unknown), dentition type (deciduous, permanent, or both), and arch (upper, lower, or both) for each radiograph under study, as well as the notation system used (Anthropology, Palmer, FDI, or Universal). The upper and lower left third molars (teeth 28 and 38) of the entire radiographic sample were analyzed, visually comparing their developmental stage to those described in the method. By selecting the developmental stage that best matches the actual one, the software provides an estimated age for the individual. This estimated age was recorded in a Microsoft Excel® spreadsheet along with the following information: radiograph number, sex, actual age, and developmental stage code. - Application of the RP Atlas Method The RP Atlas method, proposed by Sousa et al. (10), was also applied to each radiograph. In this method, an approximate age is assigned to each dental development stage. The left upper and lower third molars (teeth 28 and 38) observed in the panoramic radiographs were compared to the corresponding Atlas, and the developmental stage that best matched the actual condition was visually selected. The estimated corresponding age was recorded in the Microsoft Excel® spreadsheet, along with the relevant information and the previous assessment using the London Atlas method. - Statistical Analysis A normality assessment of the data distribution was conducted using the Shapiro-Wilk test, which indicated a skewed distribution. Based on this, Spearman's correlation test was used to evaluate the relationship between chronological age and age estimated by the two age estimation methods. A graphical assessment of this correlation was performed using Bland-Altman plots. The Lin's concordance correlation coefficient was also calculated for the overall sample, as well as for different age groups and according to patient sex. Using the legal age threshold (18 years) as a cutoff, two ROC curves were fitted to assess the ability of each method to determine adulthood. Based on the ROC curve, sensitivity, specificity, and accuracy were estimated for each cutoff point, as well as the overall area under the curve (AUC). All analyses were conducted using Stata software version 18 (StataCorp LLC, College Station, TX, USA) with a 95% significance level.

## Results

The Spearman correlation results for chronological age vs. estimated age using the London Atlas and RP Atlas methods are presented in Table 1.

Table 1

Both methods showed a high correlation with chronological age. Table 1 Spearman correlation between chronological age vs. age estimated by the London Atlas and RP Atlas methods. The London Atlas method exhibited lower mean errors compared to the RP Atlas. For both methods, the error tended to increase as age increased. Additionally, age estimation errors for females tended to be higher than for males (Table 2).

Table 2

Based on the mean absolute error, the performance of the methods was comparable, regardless of the tooth on which they were based (Table 3).

Table 3

**Figure 1:**
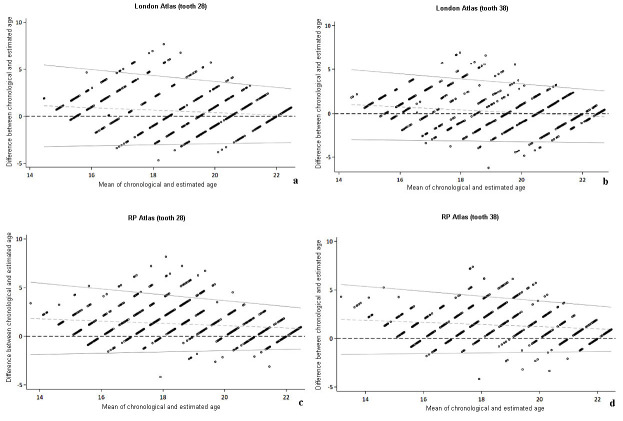
a,b,c,d) Bland-Altman plots for the London Atlas method and tooth 28 and 38, and for the RP Atlas method and teeth 28 and 38. The distribution of ages along the x-axis shows that both methods perform well in assessing age estimates for the patients included in the sample. Most observations remained within the limits of agreement.

The Bland-Altman plots illustrate the concordance analysis of the London Atlas (Fig. 1a,b) and RP Atlas (Fig. 1c,d) methods, respectively.

1

The limits of agreement tended to be wider for younger age groups compared to older ones. The Lin's concordance correlation coefficient was high (between 0.67 and 0.71) and comparable between the age estimation methods. This coefficient was higher for males compared to females. On the other hand, when evaluated according to age groups, concordance was low (Table 4).

Table 4

**Figure 2:**
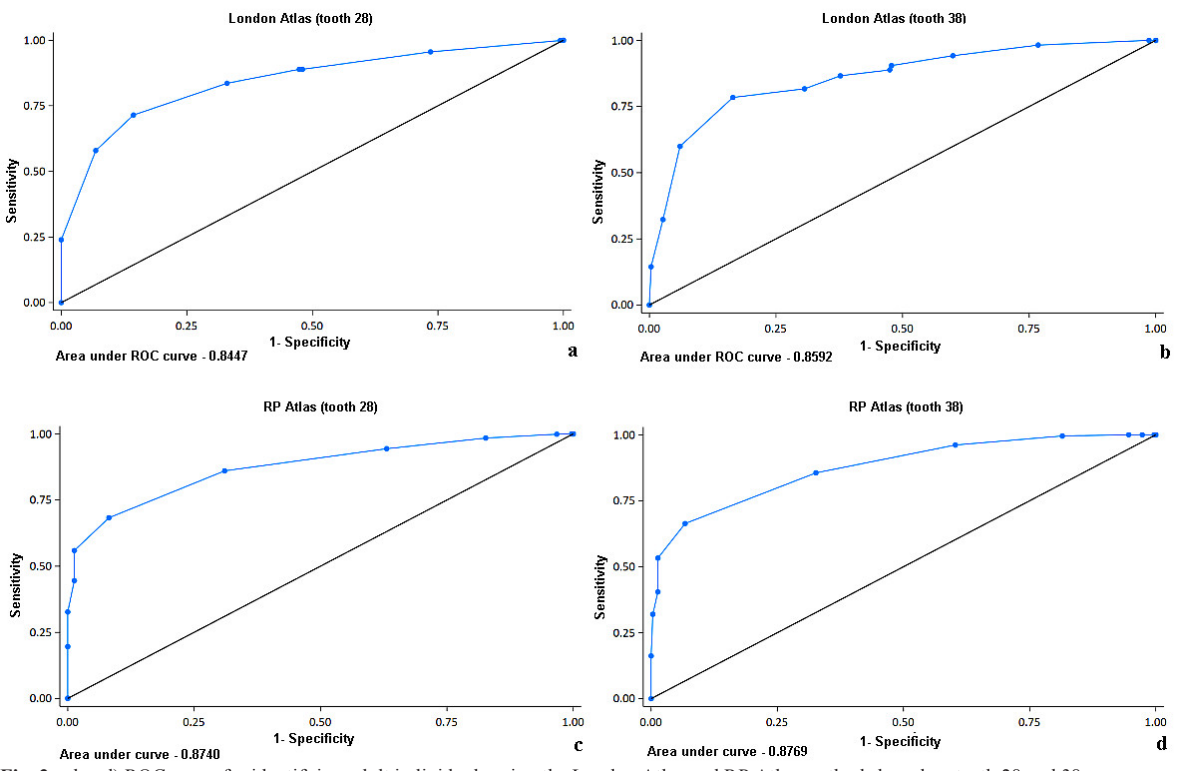
a,b,c,d) ROC curve for identifying adult individuals using the London Atlas and RP Atlas methods based on teeth 28 and 38.

Analyzing the ROC curves for the London Atlas method, it was determined that the best cutoff point for tooth 28 was 18.5 years (accuracy of 78.5%), and for tooth 38 it was 19.5 years (accuracy of 80.0%) (Fig. 2a,b).

2

The overall predictive potential of the method for both teeth was good, with AUC=0.845 (95% CI=0.821; 0.869) and AUC=0.859 (95% CI=0.835; 0.883), respectively. For the RP Atlas method, the best cutoff point was 17.0 years for both tooth 28 (accuracy of 80.8%) and tooth 38 (accuracy of 80.0%) (Fig. 2c,d). The overall predictive potential of the method for both teeth was good, with AUC=0.874 (95% CI=0.853; 0.895) and AUC=0.876 (95% CI=0.856; 0.898), respectively.

## Discussion

The present study aimed to evaluate the accuracy of the London Atlas and RP Atlas methods in age estimation and diagnosing adulthood in a sample of individuals from Southern Brazil, using images of the left third molars in panoramic radiographs. It was found that both methods showed a high correlation when evaluated against chronological age. However, the study hypothesis was rejected since the London Atlas method presented lower mean errors than the RP Atlas method. Thus, although the RP Atlas was developed in Brazil, the London Atlas proved to be a more accurate tool for age estimation in the Southern Brazilian population. This result may be attributed to the ancestral differences between the populations of the Southeastern and Southern regions of Brazil. Historical records indicate that both regions experienced significant genetic mixing and shared immigrant groups (Europeans, Africans, and East Asians). However, the South of Brazil saw a much more diverse European immigration, including Italians, Germans, Poles, and Eastern Europeans (17 , 18). The observed mean error values for the London Atlas method were less than 1, meaning any differences observed when comparing chronological age to estimated age using this method were likely to be under a 1-year difference. These results are in line with the studies by Koç, Özlek, and Talmaç (19) and Cheong et al. (20), who found mean errors of 0.83 and 0.92 years, respectively, when studying populations from Turkey and South Korea. These findings suggest that the London Atlas can estimate age with near precision in these populations. On the other hand, Namwong and Mânica (21), Dalben et al. (22), Ishwarkumar et al. (23), and Lin et al. (24) found mean errors of 1.03, 1.04, 1.02, and 1.16 years when studying the Thai, Brazilian, South African, and Chinese populations, respectively. Although still considered acceptable, these slightly higher mean errors compared to those observed in the present study highlight how the accuracy of age estimation methods can vary depending on the studied population. This underscores the necessity for validations and studies that justify the application of these methods in different contexts. It was also observed that the error in age estimation tends to increase as age increases, especially up to 21 years old. This happens because, as a person ages, dental development stabilizes, and the differences between dental development stages become less indicative of a specific age range (19 , 25). Therefore, the tendency for the error in age estimation to increase with age is not exclusive to the methods used in this study but is a finding that can be expected in most age estimation methods (6 , 19 , 24 , 26). Regarding the London Atlas method, the same pattern was found in the studies of Cheong et al. (20), Dalben et al. (22), and Zhou et al. (25), who observed greater efficacy of the method in individuals up to 20 years old, although it still proved reliable in other age groups. The error in age estimation for females tended to be higher when compared to males for both methods. It has been observed that girls generally exhibit more advanced dental maturation compared to boys, with the exception of the third molars, which develop earlier in boys (10 , 27). Karadayi et al. (27) found that this pattern was most notable in the age range of 5 to 14 years in Turkish children, usually in a single stage and not across all teeth. Sousa et al. (10), during the development of the RP Atlas method, observed that this discrepancy in dental maturation was continuous in the sample they analyzed, with girls typically being one or two stages ahead. The study by Poulsen and Sonnesen (28) reaffirms this finding by observing dental maturation in boys and girls across two birth cohorts (1969 to 1973 and 2005 to 2010). It was found that girls exhibited earlier dental maturation compared to boys in both time periods, although overall dental maturation had accelerated in children born more recently. Thus, in some populations, girls exhibit earlier dental maturation compared to boys, which can have a direct influence from sexual dimorphism, where growth is first reached by females (9). However, when considering only the third molars, girls may exhibit greater variability in development or a less consistent and uniform pattern, which increases the average error. To try to overcome these errors, the RP Atlas method was developed with distinct reference tables for females and males. Still, in this study, it was found that the error for females was slightly more pronounced than for males. However, this difference in error is not significant for either of the methods used in this study for estimating the age of this population, so the overall precision of the methods can be considered adequate for both sexes. The Bland-Altman plots showed that the limits of agreement tend to be wider for younger average ages compared to older ones, but this reduction in limits is very gradual. The distribution of ages along the x-axis shows that both methods work well for estimating the ages of the patients in the sample. Furthermore, most of the observations remained within the limits of agreement, indicating good performance of the estimation methods. The Lin's concordance coefficients were generally high (ranging from 0.67 to 0.71) and comparable between the methods, but higher for males (around 0.75) compared to females (around 0.61). On the other hand, when concordance was assessed according to age groups, it is noticeable that the concordance was low. The decrease in concordance, when assessed according to age groups, could have occurred as an artifact of the reduced sample size when restricted to the age groups, making the measure more sensitive to variations (29). The ROC curves were used to assess the potential of the methods in diagnosing the majority age of the subjects. Through this analysis, the age that maximizes the area under the curve is sought, thereby demonstrating the greatest predictive potential. For the tooth 28 analyzed using the London Atlas method, the best cutoff point for identifying individuals of legal age was 18.5 years, with an accuracy of 78.5%. For the tooth 38 analyzed with the London Atlas method, the best cutoff point was 19.5 years, with an accuracy of 80.0%. As observed in the mean error, the London Atlas method tends to overestimate the chronological age of the individual analyzed. Thus, it is understood that, if this method is used to identify individuals of legal age through teeth 28 and 38, it is more accurate to use the ages of 18.5 and 19.5 years, respectively, as the reference for majority age. For the RP Atlas method, the best cutoff point for identifying individuals of legal age, considering both tooth 28 and tooth 38, was 17.0 years, with an accuracy of 80.8% and 80.0%, respectively. Thus, the RP Atlas method presented a tendency to underestimate the chronological age of the individuals analyzed. Brazilian laws define the age of 18 as the threshold for reaching legal and criminal majority. Therefore, for relevant forensic cases, it is highly important to use specific techniques and standards with the highest possible accuracy in diagnosing the majority age for each population (6). Given the results of this study, it was found that the London Atlas method is more appropriate for diagnosing legal majority in the studied sample, as it is preferable for methods to overestimate the age to avoid diagnosing majority in individuals who are still minors. With the RP Atlas results indicating a cutoff for majority age of 17.0 years for teeth 28 and 38, there is a greater risk of minors being misclassified as adults. Thus, the hypothesis raised in this study was rejected, as, although developed in Brazil, the RP Atlas underestimates the age of individuals in the southern Brazilian sample studied, which is not favorable in legal processes for diagnosing majority age. All atlas-based methodologies have limitations. The illustrated tables are not capable of representing all cases. Overlapping stages of maturity, the lack of differentiation by sex (resulting in a high degree of variability), and the possibility of lack of agreement between observers may lead to higher error rates compared to other techniques for assessing dental development. Therefore, there is a need for further investigations to adjust and improve the applicability of these methods in different populations and age groups, especially in individuals over 21 years of age. Research that applies these methods can provide a foundation for adjustments to the Atlases and their application in diverse subpopulations, and even serve as the basis for the establishment of an international data repository based on the various available Atlases for different ethnic subpopulations. Both age estimation methods proved to be suitable for the studied sample, with the London Atlas showing greater accuracy, as evidenced by lower mean errors compared to the RP Atlas. Greater imprecision was observed for females in both methods. Accuracy also tended to decrease in age groups older than 21 years. Both methods were effective in diagnosing the majority. In this regard, the London Atlas stands out for its tendency to overestimate age, a characteristic that can be advantageous in legal situations that require greater caution in the diagnosis of majority age. So, the London Atlas method is the most recommended for age estimation in the studied population, being preferable in contexts where precision and caution in the diagnosis of majority age are essential. One relevant aspect observed in the practical application of the RP Atlas was the occasional difficulty experienced by operators in visually matching tooth development to the stages presented in the reference charts. This issue was mainly due to graphical limitations and the overlapping of age ranges across adjacent stages. Improvements to the graphical clarity and structure of the developmental stages in the RP Atlas could enhance its usability and reduce operator-dependent variability. Furthermore, adjustments in the RP Atlas could also include sex-specific or ancestry-informed developmental stages, enhancing its adaptability to specific demographic groups. Expanding the RP Atlas beyond third molars, incorporating additional teeth or developmental indicators, may also improve accuracy in cases where third molars are absent or impacted. Finally, beyond forensic purposes, accurate age estimation has important clinical implications. Dental age estimation methods can contribute to treatment planning in orthodontics and pediatric dentistry, where age-related development is crucial for determining appropriate interventions. Additionally, such tools may assist in monitoring delayed or advanced dental development in adolescents, supporting broader healthcare decisions. Other clinical fields can also benefit from age estimation, such as implantology and oral rehabilitation, where the evaluation of skeletal and dental maturity is essential before initiating definitive treatments. In cases of syndromic conditions or systemic diseases that affect growth and development, dental age estimation may aid in early diagnosis or longitudinal follow-up. These applications reinforce the interdisciplinary value of dental age estimation and its role in improving patient outcomes beyond legal and forensic contexts.

## Figures and Tables

**Table 1:** Spearman correlation between chronological age vs. age estimated by the London Atlas and RP Atlas methods.

	Chronological age	London Atlas (28)	London Atlas (38)	RP Atlas (28)	RP Altas (38)
Chronological age	1.000				
London Atlas (28)	0.722	1.000			
London Atlas (38)	0.736	0.847	1.000		
RP Atlas (28)	0.801	0.908	0.832	1.000	
RP Altas (38)	0.798	0.847	0.914	0.903	1.000

1

**Table 2:** Analysis of mean error between estimated age through the London Atlas and RP Atlas methods and chronological age for the overall sample and according to age groups and sex.

	London Atlas		RP Atlas	
	Tooth 28	Tooth 38	Tooth 28	Tooth 38
Overall	0.52 (0.40; 0.64)	0.23 (0.12; 0.35)	1.25 (1.15; 1.35)	1.37 (1.28; 1.47)
Age group				
15-	-0.11 (-0.36; 0.14)	-0.29 (-0.61; 0.03)	0.29 (0.06; 0.51)	0.48 (0.17; 0.78)
16-	0.06 (-0.25; 0.36)	-0.02 (-0.32; 0.28)	0.77 (0.58; 0.95)	0.82 (0.64; 1.01)
17-	-0.46 (-0.84; -0.09)	-0.59 (-0.97; -0.21)	0.89 (0.66; 1.12)	1.00 (0.75; 1.24)
18-	0.26 (-0.13; 0.66)	0.07 (-0.34; 0.47)	1.47 (1.25; 1.69)	1.46 (1.24; 1.69)
19-	0.03 (-0.28; 0.35)	-0.16 (-0.44; 0.13)	1.55 (1.31; 1.80)	1.63 (1.42; 1.84)
20-	0.97 (0.71; 1.23)	0.42 (0.16; 0.68)	1.51 (1.24; 1.79)	1.72 (1.46; 1.99)
21-	1.32 (1.04; 1.61)	0.91 (0.62; 1.20)	1.64 (1.32; 1.96)	1.76 (1.45; 2.07)
22-	1.50 (1.23; 1.76)	1.11 (0.88; 1.34)	1.35 (1.08; 1.63)	1.50 (1.22; 1.77)
Sex				
Male	0.20 (0.04; 0.35)	0.28 (0.13; 0.43)	0.89 (0.76; 1.03)	1.06 (0.93; 1.18)
Female	0.75 (0.59; 0.91)	0.20 (0.03; 0.36)	1.51 (1.37; 1.64)	1.60 (1.47; 1.74)

2

**Table 3:** Analysis of mean absolute error between the estimated age using the London Atlas and RP Atlas methods and the chronological age for the overall sample, as well as according to age group and sex. The mean absolute errors were higher in females compared to males. The increase in mean absolute errors with age was observed in some cases, mainly up to 21 years old.

	London Atlas		RP Atlas	
	Tooth 28	Tooth 38	Tooth 28	Tooth 38
Overall	1.40 (1.32; 1.48)	1.41 (1.34; 1.49)	1.45 (1.36; 1.53)	1.56 (1.47; 1.65)
Age group				
15-	0.89 (0.71; 1.06)	1.18 (0.97; 1.39)	0.93 (0.79; 1.07)	1.16 (0.95; 1.36)
16-	1.43 (1.29; 1.58)	1.37 (1.22; 1.53)	1.00 (0.86; 1.15)	1.04 (0.89; 1.18)
17-	1.77 (1.61; 1.93)	1.75 (1.56; 1.95)	1.14 (0.96; 1.32)	1.23 (1.04; 1.43)
18-	1.76 (1.53; 1.99)	1.93 (1.74; 2.11)	1.61 (1.43; 1.80)	1.66 (1.50; 1.83)
19-	1.47 (1.27; 1.67)	1.38 (1.21; 1.55)	1.77 (1.56; 1.97)	1.75 (1.58; 1.93)
20-	1.28 (1.05; 1.50)	1.22 (1.03; 1.41)	1.83 (1.61; 2.06)	1.93 (1.70; 2.16)
21-	1.53 (1.27; 1.78)	1.40 (1.17; 1.64)	1.77 (1.47; 2.07)	1.86 (1.56; 2.16)
22-	1.50 (1.23; 1.76)	1.13 (0.90; 1.36)	1.35 (1.08; 1.63)	1.50 (1.22; 1.77)
Sex				
Male	1.25 (1.14; 1.35)	1.24 (1.15; 1.34)	1.23 (1.13; 1.33)	1.29 (1.18; 1.39)
Female	1.60 (1.48; 1.71)	1.54 (1.43; 1.64)	1.67 (1.55; 1.79)	1.78 (1.66; 1.90)

3

**Table 4:** Analysis of the Lin concordance coefficient comparing the estimated age by the London Atlas and RP Atlas methods to chronological age for the overall sample and according to age groups and sex.

	London Atlas		RP Atlas	
	Tooth 28	Tooth 38	Tooth 28	Tooth 38
Overall	0.67 (0.64; 0.71)	0.71 (0.68; 0.74)	0.68 (0.65; 0.71)	0.67 (0.64; 0.70)
Age group				
15-	0.12 (0.07; 0.16)	0.10 (0.06; 0.14)	0.14 (0.09; 0.19)	0.09 (0.05; 0.14)
16-	0.15 (0.12; 0.18)	0.16 (0.14; 0.19)	0.17 (0.13; 0.21)	0.17 (0.13; 0.21)
17-	0.11 (0.08; 0.14)	0.13 (0.11; 0.15)	0.12 (0.09; 0.16)	0.13 (0.09; 0.16)
18-	0.09 (0.06; 0.11)	0.09 (0.07; 0.11)	0.06 (0.04; 0.09)	0.07 (0.05; 0.10)
19-	0.10 (0.08; 0.13)	0.10 (0.07; 0.12)	0.08 (0.06; 0.10)	0.06 (0.04; 0.08)
20-	0.11 (0.09; 0.14)	0.13 (0.10; 0.16)	0.08 (0.06; 0.10)	0.08 (0.06; 0.09)
21-	0.08 (0.05; 0.10)	0.09 (0.06; 0.11)	0.06 (0.04; 0.08)	0.07 (0.05; 0.08)
22-	0.07 (0.04; 0.10)	0.08 (0.05; 0.12)	0.08 (0.05; 0.11)	0.07 (0.05; 0.10)
Sex				
Male	0.75 (0.71; 0.79)	0.77 (0.73; 0.81)	0.78 (0.74; 0.81)	0.76 (0.72; 0.80)
Female	0.62 (0.57; 0.67)	0.66 (0.62; 0.71)	0.63 (0.59; 0.67)	0.61 (0.56; 0.65)

4

## Data Availability

The datasets used and/or analyzed during the current study are available from the corresponding author.
